# The Meaning of Adherence When Behavioral Risk Patterns Vary: Obscured Use- and Method-Effectiveness in HIV-Prevention Trials

**DOI:** 10.1371/journal.pone.0044029

**Published:** 2012-08-31

**Authors:** Marijn de Bruin, Wolfgang Viechtbauer

**Affiliations:** 1 Department of Communication Science, Wageningen University, Wageningen, The Netherlands; 2 Department of Psychiatry and Psychology, School for Mental Health and Neuroscience, Faculty of Health, Medicine, and Life Sciences, Maastricht University, Maastricht, The Netherlands; Duke University School of Medicine, United States of America

## Abstract

**Background:**

Recently promising trials of innovative biomedical approaches to prevent HIV transmission have been reported. Participants' non-adherence to the prevention methods complicates the analyses and interpretation of trial results. The influence of variable sexual behaviors within and between participants of trials further obscures matters. Current methodological and statistical approaches in HIV-prevention studies, as well as ongoing debates on contradictory trial results, may fail to accurately address these topics.

**Methodology/Principal Findings:**

Through developing a cumulative probability model of infection within HIV prevention trials, we demonstrate how adherence and sexual behavior patterns impact the overall estimate of effectiveness, the effectiveness of prevention methods as a function of adherence, and conclusions about methods' true effectiveness. Applying the model to summary-level data from the CAPRISA trial, we observe markedly different values for the true method effectiveness of the microbicide, and show that if the gel would have been tested among women with slightly different sexual behavior patterns, conclusions might well have been that the gel is not effective.

**Conclusions/Significance:**

Relative risk and adherence analyses in HIV prevention trials overlook the complex interplay between adherence and sexual behavior patterns. Consequently, they may not provide accurate estimates of use- and method-effectiveness. Moreover, trial conclusions are contingent upon the predominant sexual behavior pattern of participants and cannot be directly generalized to other contexts. We recommend researchers to (re)examine their data and use the cumulative probability model to estimate the true method effectiveness, which might contribute to resolving current questions about contradictory trial results. Moreover, we suggest taking into account the issues raised in the design of future trials and in population models estimating the impact of large-scale dissemination of prevention methods. Comprehension of the topics described will help readers to better interpret (apparently contradictory) trial outcomes.

## Introduction

Despite recent indications that the spread of HIV is declining in several parts of the world, HIV remains one of the most severe pandemics in human history, with an estimated 33.3 million people currently living with the disease [1]. New approaches to prevent HIV transmission can therefore have an impact on the lives of millions. Major efforts have been directed at the identification of safe and effective pre-exposure prophylaxes (PrEP), with alternately spectacular and disappointing results in recent years [2], [3], [4], [5]. The results from the CAPRISA 004 trial reported by Karim and colleagues in the summer of 2010 were a breakthrough, since it was the first trial to show that antiretroviral microbicides can be a promising new tool for HIV-prevention [3]. Specifically, the CAPRISA team observed a 39% lower HIV incidence rate among women receiving a vaginal tenofovir gel compared to those receiving a placebo, a statistically significant and clinically meaningful effect.

A series of prior studies had failed to show a protective effect of microbicides [6], [7]. However, as Weiss and colleagues (2008) have pointed out, not finding an effect does not necessarily imply that the microbicide studied is not efficacious [8]. Inconsistent or incorrect use of microbicides is a common problem, with an estimated 30% of coital acts not being covered by the use of the gel [7]. Clearly, more consistent use of PrEP should result in better preventive effects if the method is in fact protective. This is exactly what the CAPRISA team observed in their study, where high adherers (i.e., women who used the gel in >80% of the sexual encounters) had a 54% lower HIV-incidence compared to 38% for medium (50–80% gel use) and 28% for low adherers (<50% gel use) [3]. Such adherence analyses are essential, since they can reveal the true method effectiveness, information that is vital for directing future research efforts (i.e., do better microbicides need to be developed or should efforts be directed at designing effective adherence interventions and efficient gel dissemination strategies?).

Although PrEP trials have paid attention to the role of adherence, and even though the variable effectiveness of the tenofovir gel in the CAPRISA trial was found to be related to participants’ adherence in the expected direction, the studies conducted so far seem to suffer from several important methodological and statistical limitations. Consequently, trials may have suggested that a PrEP is not effective when in fact it is, or may have produced incorrect estimates of the effectiveness of PrEP under variable adherence conditions. In this paper, we describe these concerns, using the CAPRISA trial as an illustration, and discuss a framework for including adherence and other potential effect modifiers in a more meaningful manner in the analyses. Through mathematical modeling, we demonstrate that applying this framework may result in markedly different conclusions about the overall treatment effectiveness, the effectiveness under variable adherence levels, as well as the true method effectiveness of the prevention method tested. Although some of the issues discussed will need to be addressed in future research, we recommend that authors of previous PrEP trials to re-examine their primary and adherence-related analyses.

For the purpose of clarity in terminology and an illustration based on CAPRISA data, we will focus on vaginal microbicide studies hereafter and return to PrEP trials in general in the Discussion.

### Making Sense of Adherence Percentages

In this section, we describe the concept of adherence and then discuss several issues when trying to relate adherence to an HIV prevention method to absolute HIV infection risk. In the next section, we explain and demonstrate how these issues also impact relative risks of infection in double-blind, placebo controlled HIV prevention trials.

In the context of medication studies, adherence has been defined as “the extent to which patients' actual history of drug administration corresponds to the prescribed regimen” [9]. Adherence is usually computed by dividing the number of medication doses taken (or taken within the prescribed time interval; the numerator) by the number of doses prescribed (the denominator), multiplied by 100 to arrive at an adherence percentage [10]. Hence, when two patients are prescribed a once-daily regimen and have 70% adherence after 100 days, it means that both patients have omitted the taking of 30 pills. Assuming that other potential treatment effect modifiers are comparable between patients (e.g., pharmacokinetics and pharmacodynamics; cf. [11], [12]) and that patterns of missed doses are similar, 70% adherence is then considered to represent a similar absolute risk of disease progression for both patients (i.e., 30 days without therapeutic coverage). An important assumption underlying this adherence percentage is that each day without therapeutic coverage entails the same risk of disease progression (i.e., missing a pill on Sunday encompasses the same risk as missing a pill on Tuesday). In medication adherence studies, the main challenge in studying this topic is the accurate measurement of patients' pill intake behavior over time and analysis of the impact of different adherence patterns [11].

In microbicide trials the situation is considerably more complex. Typically adherence is computed by dividing the number of gel applicators used over a certain time period (e.g., 1 month) by the number of coital acts during that period. Hence, first, researchers need to obtain reliable data on both the numerator (the number of gel applicators used correctly) and the denominator (the number of coital acts). There are well-recognized challenges related to the reliable measurement of such intimate data on which others have written excellent work [13], [14], [15], [16].

Second, current computations of adherence percentages in microbicide trials are based on the assumption that each sexual encounter entails the same risk of obtaining HIV. However, although a reliable behavioral proxy for HIV infection has not yet been established [17], it is known that infection risk depends on a variety of factors including the type of sexual practice [18], whether condoms were used [19], whether the male partner is circumcised [20], [21], and the presence of other sexually transmitted diseases (e.g., [22]). Since, depending on these parameters, the riskiness of a sexual encounter can vary considerably, adherence can only be meaningfully incorporated into the analysis if one treats gel use over the set of sexual encounters that comprise risky behaviors differently than gel use over (non- or) less-risky encounters.

The third issue pertains to the fact that similar adherence percentages in HIV prevention trials do not represent the same degree of riskiness for different people. Whereas 70% adherence represents 30 days without therapeutic coverage for patients using a once-daily pill regimen for 100 days, in HIV prevention studies some women may have had 10 sexual encounters while others may have had 100. So what then does, for example, 70% gel adherence imply in terms of potential risk reduction by using the gel? For women who had 10 sexual encounters, it meant having approximately 3 events “without gel coverage”. For women who had 100 sexual encounters, it meant having 30 events without the protection of the gel. In other words, the adherence percentage does not represent the same absolute risk across different risk behavior patterns (i.e., between women or within women over time).

Fourth, an additional element that must be considered in the context of microbicide trials is how the number of (high- and low-risk) contacts are distributed over a given number of partners. In particular, the risk of HIV infection differs whether a woman has 100 contacts with a single partner, 10 contacts with 10 different partners, or one contact with 100 different partners [23]. Therefore, when analyzing the influence of adherence, one must not only account for the riskiness of the contacts and the total number of contacts, but also for the number of partners that the women have intercourse with during the course of the trial.

Finally, HIV-prevention trials can take several years to complete, with end points for participants being either an HIV infection during the study or no seroconversion at the end of the study. HIV-tests are typically conducted every 1–3 months in these trials. Therefore, researchers can estimate the time window in which the HIV infection must have occurred (e.g., the previous 3 months). Since sexual behavior and adherence may vary over time and patients may stop using the gel periodically or even completely (which is also common within medication trials; see Blaschke et al., 2012) [11], capturing the relevant risk behaviors and data on gel adherence over these time periods, as compared with the time periods that did not lead to seroconversion, should provide considerably more accurate estimates of HIV-risk behavior patterns and (non)adherence, and thus the effectiveness of the gel under variable adherence conditions. At present, studies typically use the average or median score over the whole study period for some parameters (e.g., adherence) and baseline values for others (e.g., number of partners in the past month).

### From Absolute to Relative Risks

We assume that two of the issues discussed above, namely the need for reliable measurements and the summarizing of these data in meaningful time-windows preceding HIV-tests, speak for themselves. What is less evident, however, is that behaviors that affect the absolute infection risk (i.e., the riskiness of sexual encounters, the frequency of these encounters, the number of partners) can also affect relative risks, since randomization in HIV-prevention trials should lead to such behavior patterns being equally distributed over the treatment and placebo conditions.

First, consider the point that, in order to obtain an accurate estimate of the relation between adherence and the relative risk on becoming infected, one needs to control for the total number of risky encounters over which adherence has been computed. We will illustrate this point using a cumulative probability (or Bernoulli-process) model of HIV infection [23]–[26]. The cumulative probability model is a simple mathematical model based on the rules of probability that describes how the probability of infection accumulates when risk factors, such as number of contacts or the number of partners, increase. An overview of the mathematical symbols and their meaning used throughout this paper is provided in Table 1.

**Table 1:** Symbols and abbreviations used in the cumulative probability model and their interpretation.

Symbol	Interpretation
α	per-contact HIV infection probability for a single unprotected (i.e., without condoms) sexual encounter with an HIV-positive partner
θ	per-contact relative HIV infection probability for a single unprotected (i.e., without condoms) sexual encounter with an HIV-positive partner when using the gel (therefore 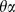 reflects the per-contact risk of infection when using the gel)
*_n_*	number of unprotected (i.e., without condoms) encounters (per partner)
λ_n_	proportion of unprotected (i.e., without condoms) encounters where the gel is used
*_k_*	number of protected (i.e., with condoms) encounters (per partner)
λ_k_	proportion of protected (i.e., with condoms) encounters where the gel is used
ε	probability of a condom failing to provide proper protection
π	prevalence of HIV in the population
*_m_*	number of partners
*_Pc_*	cumulative risk of infection in the control group implied by the model based on the behavioral pattern of the women
*_PT_*	cumulative risk of infection in the intervention group implied by the model based on the behavioral pattern of the women
*_RR = PT /Pc_*	cumulative relative risk of infection implied by the model for women in the intervention group compared to women in the control group

Let 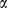 denote the per-contact probability of an HIV infection for a single unprotected (i.e., without condoms) encounter with an HIV-positive partner. Then the cumulative risk of infection for a total of 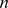 unprotected contacts with an HIV-positive partner for women in the control group is obtained by subtracting the risk of never becoming infected in any of the 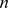 contacts (i.e., 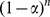) from 1, yielding
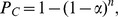
the probability of seroconversion in one of the 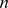 encounters.

For women in the treatment group, the per-contact risk of infection is reduced by a certain amount when using the gel. We will let 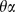 denote this reduced per-contact infection risk, so that 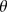 reflects the true relative risk (i.e., the gel's true effectiveness for reducing the per-contact infection risk). Moreover, let 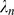 denote the proportion of encounters unprotected by condoms where the gel is used. Then the cumulative risk of infection in the treatment group is given by subtracting the product of the probabilities of not becoming infected on any of the occasions where the gel was used (i.e., 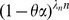) and where it was not used (i.e., 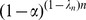) from 1, yielding
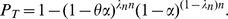



**Figure 1:**
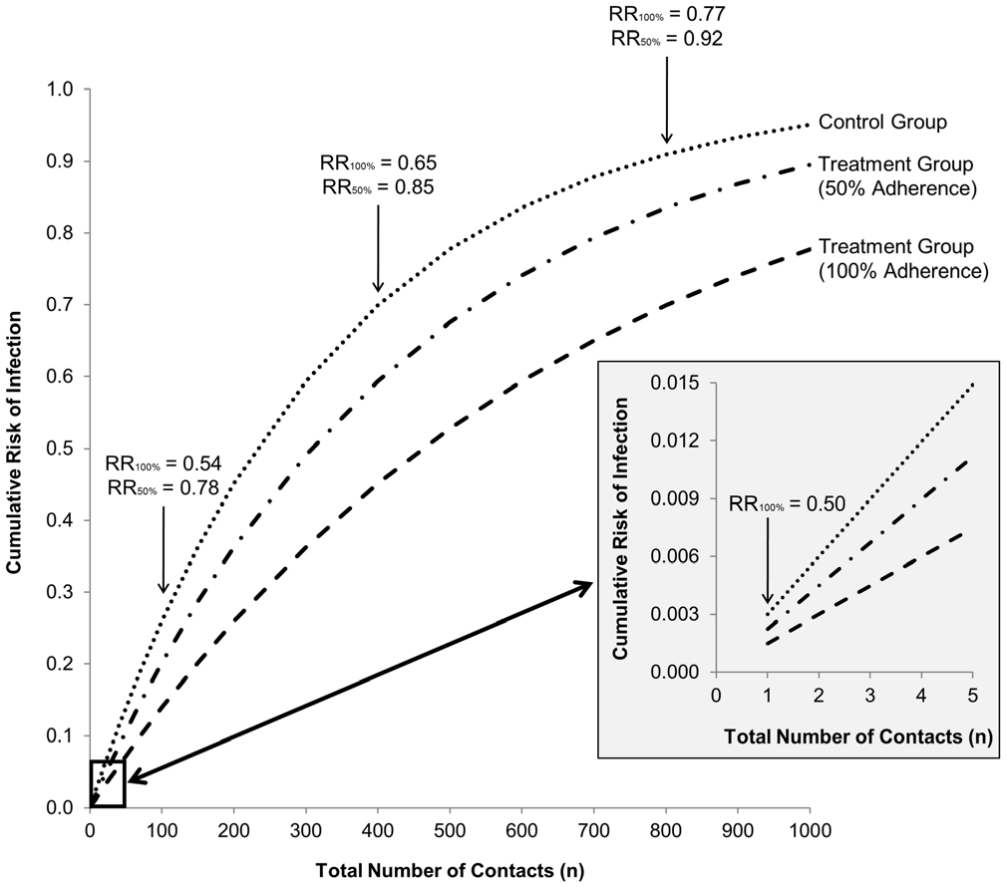
Cumulative risk of infection as a function of the number of unprotected contacts (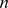) with an HIV-infected partner for women in the control and treatment group for different levels of adherence to the intervention gel (RR_100%_ and RR_50%_ indicate the relative risk of infection for 100% and 50% adherence to the gel).

Figure 1 displays the relation between the number of sexual encounters without condoms (on the x-axis) and the cumulative risk of infection for control participants (top line) and intervention participants with 50% and 100% adherence (middle and bottom line, respectively) on the y-axis, assuming a per-contact infection risk of 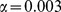 (based on the recent meta-analysis by Boily, 2009) [18] and an assumed efficacy of a microbicide gel of 50% (i.e., 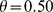). The figure inset shows a close-up for small *n*. In addition, the relative infection risk, 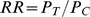, is indicated when comparing women in the treatment group with women in the control group for a given number of *n* and assuming either 50% or 100% adherence.

As can be seen, with only 1 unsafe contact during which the gel was used, the relative risk of infection in the treatment group is 0.50 (i.e., the gel's true effectiveness). With an increasing number of contacts, the infection risk increases non-linearly, with a relative risk of 0.54 after 100 encounters for 100% adherence and a relative risk of 0.78 for 50% adherence. As the number of risky contacts increases further (the range used is larger than what is typically observed in a trial, however, it is not unrealistic from a life-time perspective), the absolute infection risk in all groups approaches 1 and so does the relative risk. Therefore, Figure 1 shows that coital frequency has a direct impact on the cumulative relative risk, regardless of the level of adherence (i.e., the apparent effectiveness of the gel decreases with increasing coital frequency). Moreover, the impact of adherence on the relative risk (i.e., the difference in the relative risk for 50% and 100% adherence) also depends on whether one focuses on women with 1, 100, 400, or 800 contacts. For example, for 100 contacts, 50% adherence implies a 0.78/0.54 = 1.44 times higher infection risk than 100% adherence. However, for 400 contacts, the difference between 50% and 100% adherence is smaller, leading to a 0.85/0.65 = 1.31 times higher risk of infection. Figure 1 thus shows how both the cumulative relative risk as well as the relation between adherence and relative risk, due to the accumulation of risk over time in both groups, depends non-linearly on the number of risky contacts.

Second, consider the point that, in order to obtain an accurate estimate of the relation between adherence and the relative risk of becoming infected, one needs to control for behaviors that can impact the per-contact infection risk. In microbicide trials, the most important (i.e., most common and protective) behavior among sexually active women, on which data is also typically collected, is condom use. Let *k* denote the number of condom-protected contacts and the chance of a condom failing to provide proper protection (e.g., due to incorrect use, slippage, or breakage). We will assume a value of 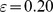 based on the results from a meta-analysis on condom effectiveness for reducing HIV transmission [19]. The cumulative risk of infection in the control and treatment group when always using condoms is then given by
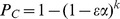
and



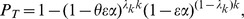
respectively, where 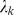 now denotes the proportion of condom-protected encounters where the gel is used. For a single encounter, the relative infection risk is then again equal to 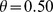. However, since the infection risk now accumulates more slowly as the number of condom-protected contacts increases (i.e., the 3 lines in Figure 1 would have much flatter slopes), relative infection risks can be markedly different for women using condoms and not using condoms, despite similar adherence levels and number of sexual encounters. For example, for 400 encounters protected by condoms, the relative risk is now equal to 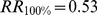 for 100% adherence and 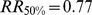 for 50% adherence. Not only do these relative risks differ from those we found earlier for the same number unprotected encounters (i.e., 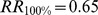 and 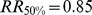; see Figure 1), but the ratio of these relative risks also differs (i.e., 50% adherence implies a 0.85/0.65 = 1.31 times higher risk of infection compared to 100% adherence for 400 unprotected contacts; the same number of protected contacts implies that 50% adherence is 0.77/0.53 = 1.45 times more risky). Consequently, not only the number of contacts, but also the riskiness of the contacts must be considered when interpreting the cumulative relative risk and when examining the influence of adherence on the relative risk of infection. In practice, this could be accomplished by calculating separate adherence percentages for unprotected and protected contacts.

Besides the total number of contacts and the riskiness of the contacts, a third factor to consider is the number of partners. For this, the actual prevalence of HIV in the population (in this case, the population of men with whom women may have sexual contacts) needs to be incorporated into the computations. Based on recent UNAIDS figures [1], we will assume a prevalence of 20% (i.e., 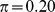) to represent a high prevalence region in Sub-Saharan Africa. Now consider women in the control group who have 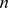 unprotected contacts per partner with a total of 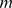 different partners (for a total of 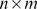 unprotected contacts). Based on the cumulative probability model of HIV infection, their infection risk is given by
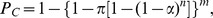
where 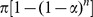 is the joint probability that a particular partner is HIV-positive and the risk that seroconversion occurs in at least one of the 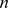 unprotected contacts with that partner (hence 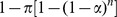 is the probability that no seroconversion occurs with that partner) and 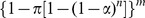 denotes the probability of no seroconversion with any of the 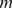 partners. On the other hand, for women in the treatment group, the cumulative risk is equal to



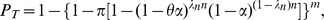
which incorporates the reduced per-contact infection risk for encounters where the gel is actually used.

Several important points can be illustrated based on these equations. First, for a given number of total contacts, the absolute infection risk increases as the number of partners increases. For example, while 400 unprotected contacts with a single partner implies a 0.14 risk of infection for women in the control group, 40 contacts per partner with a total of 10 different partners implies a cumulative risk of 0.21. Therefore, all else equal, a higher number of partners increases one's risk of being exposed to HIV and hence one's risk of becoming infected (see also [17]). Second, the *relative* infection risk between intervention and control participants also depends on the number of partners. For example, assuming that the women in the treatment group are 100% adherent, the relative risk is 0.65 for women with 400 unprotected contacts with a single partner while 40 contacts per partner with a total of 10 partners yields a relative risk of 0.54. Hence, the differences in absolute infection risk carry over to the relative risk. Finally, also the impact of adherence on the relative risk depends on the number of partners. For example, for 50% (instead of 100%) adherence, the relative risks for the two behavior patterns described above are 0.85 and 0.78, respectively. Therefore, while the relative risk of infection is 0.85/0.65 = 1.31 times greater for 50% as opposed to 100% adherence for women with 400 contacts with a single partner, the relative risk is 0.78/0.54 = 1.44 times greater for women who have 40 contacts with 10 partners each.

In HIV prevention trials, the factors discussed above all act simultaneously. However, although microbicide trials tend to collect data on adherence, number of partners, coital frequency, condom use, and sometimes on gel adherence over condom and non-condom use events separately, neither their overall analyses of the relative risk nor any secondary adherence analyses take these factors into account. Our modeling suggests, however, that this has most likely resulted in incorrect estimates of the overall treatment effectiveness, the effectiveness of gels under different adherence levels, as well as the true method effectiveness of microbicides.

In the next section, a cumulative probability model is described that incorporates all these different elements. This model can be used with aggregate or individual-level trial data to evaluate the effectiveness of a biomedical prevention measure that does consider these potentially important effect modifiers. We use the model to illustrate (a) how the true method effectiveness of prevention measures can be estimated while taking variable risk behavior patterns into account, (b) the impact of different behavioral risk patterns on conclusions about treatment effectiveness under variable adherence conditions, and (c) how different, plausible behavioral patterns in the study population can lead to very different conclusions about overall treatment effectiveness. We will use data from the CAPRISA trial to illustrate these points.

### Integrated Cumulative Model and Application to the CAPRISA Trial

In a typical microbicide trial, women are randomly assigned to either the actual treatment group or a placebo condition. At regular intervals (e.g., once per month), the number of sexual partners, the number of sexual contacts per partner, condom usage, and gel adherence are assessed. For the sake of the argument, we assume that these measurements are reliable and valid. As above, *m* denotes the total number of partners for a particular woman over the entire study period, *n* the number of sexual encounters per partner without the protection of condoms, and *k* the number of encounters with condoms (note that for simplicity, we assume that *n* and *k* are the same for each partner, although one could easily extend the calculations to situations where this assumption does not hold. The main conclusions, however, would remain unchanged). All of these variables exert an influence on the risk of infection, which accumulates with increasing values of *m*, *n*, and *k*. The particular combination of *m*, *n*, and *k* denotes a certain behavior pattern (e.g., monogamy, polygamy).

For women in the control group, the cumulative risk of infection for a particular behavior pattern is then given by
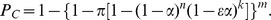



Applying this model to the CAPRISA trial requires obtaining sample-specific data, since HIV prevalence, coital frequency, condom use, and number of partners can vary considerably between regions and populations. Table 2 lists the values that were used for the various variables in the model. The source and/or derivation of these values is discussed in the remainder of this section.

**Table 2:** Values used for the cumulative probability model for the CAPRISA trial.

Variable	Value
α	0.015
θ	0.32
*n*	9
λ*_n_*	0.44
*k*	36
λ*_k_*	0.78
ε	0.20
π	0.32
*m*	2
*_Pc_*	0.134
*_PT_*	0.085

Recent figures indicate that, of all regions in South Africa, HIV prevalence rates are among the highest in the KwaZulu-Natal province, where the trial was conducted [27]. For adult resident males in the age range of 20–39, Welz and colleagues (2007) observed a prevalence of 32%, so we set 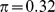 [28]. As before, we assume that the chance of a condom failing is 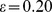. On average, women in the CAPRISA trial had 90 sex acts, 18 without condoms (i.e., risky contacts) and 72 with (i.e., less-risky contacts) [3]. Based on the baseline CAPRISA data, we assume that the women had on average 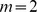 partners during the 18 months, which corresponds to an average of 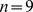 contacts per partner without condoms and 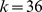 contacts with condoms (note that in sensitivity analyses the conclusions remained unchanged when changing the number of partners to 1, 3, or 4). Using these numbers in the equation above and setting the per-contact infection risk to 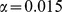, we obtain an average cumulative risk of
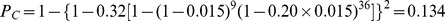
(corresponding closely to the 60 HIV infections observed among the 444 women in the placebo group). The value of 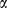 used here is quite high (cf. [18], [29]), but so was the infection risk observed in the control group (i.e., 9.1 cases per 100 woman-years) *despite* low levels of reported sexual risk behavior and STI screening and treatment at baseline. We will return to this issue in the discussion.

As discussed earlier, gel use should reduce the per-contact infection risk in the treatment group. Using 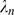 again to denote the proportion of sexual encounters without condoms where the gel was properly used (i.e., high-risk contacts) and 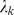 the proportion of encounters with condoms where the gel was properly used (i.e., low-risk contacts), the cumulative risk of infection for women in the treatment group is then equal to



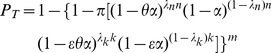
.

In the CAPRISA trial, an average of approximately 70% of the sex acts were covered by the prescribed two doses of gel, but it is unknown how adherence was distributed over high- and low-risk contacts. The results from other microbicide trials that did report gel adherence over condom and non-condom use contacts consistently show higher gel adherence levels (i.e., approximately 1.7 times higher) on encounters where condoms are used (e.g., [30]–[32]). We therefore assume that 8 out of the 18 high-risk contacts (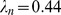) and 56 out of the 72 low-risk contacts (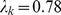) were covered by gel use, giving an overall adherence of approximately 70% (and a value of 0.78/0.44 = 1.77 for the ratio of the adherence for low- versus high-risk contacts). Using these estimates, the true method effectiveness (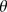) can be computed (via a root-finding algorithm or, more easily, by trial-and-error by plugging increasing values of 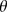 into the equation) that produces the average cumulative risk of 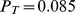, which corresponds to the 38 HIV infections observed among the 445 women in the treatment group. One can easily verify that with 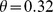, we obtain
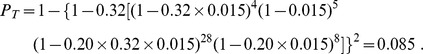



The value 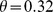 corresponds to a 68% relative risk reduction, which, based on the data available and the model assumptions, can be regarded as the true method effectiveness of the tenofovir gel (different from the 54% reported by CAPRISA for the high-adherent women). This per-contact risk reduction needs to be clearly distinguished from the ratio of the cumulative risks (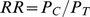), which denotes the relative risk based on the behavior pattern the women engaged in during the study (i.e., 0.63 in our model, deviating slightly from the 0.61 reported by the CAPRISA trial due to rounding errors). This application of the cumulative probability model thus illustrates how the true effectiveness of an HIV-prevention method can be identified, accounting for the unique adherence and sexual risk behavior patterns among trial participants, which can have a notable impact on trial conclusions.

### Effects of Variable Risk Behaviors Patterns on Trial Outcomes

With the true method effectiveness estimated and the other parameters as well as the outcomes fitting the CAPRISA data, the model will now be used to illustrate the impact of various plausible risk behavior patterns on trial conclusions about the overall effectiveness of an HIV prevention method and its effectiveness under variable adherence levels. Note that having to estimate some parameters in the model to fit the CAPRISA data based on other prevention trials and meta-analyses affects our confidence in the estimate of θ, but not in any of the principles illustrated.

First, consider the sexual behavioral patterns monogamy (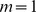 partner with 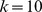 protected followed by 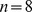 unprotected contacts), serial monogamy (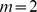 partners with 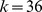 protected and 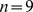 unprotected contacts per partner), and promiscuity but with high levels of condom use (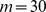 partners with 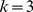 protected and 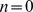 unprotected contacts per partner). While we assume that women with these different behavioral patterns all have a total of 90 contacts, the relative infection risks are very different, even when keeping 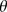 and adherence constant at the values calculated earlier. For monogamous women, the 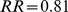, while for the serial monogamy and promiscuous behavior patterns, the relative risks are 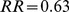 and 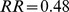, respectively. Hence, depending on the dominant risk behavior patterns of women in a trial, very different relative risks may be observed. The importance of this point should not be underestimated: if the tenofovir gel would have been tested among the women in the trial by Skoler-Karpoff and colleagues [33], with somewhat higher coital frequency and lower condom use percentages, the relative risk would have been between 0.79 (assuming an average of 1 partner) and 0.74 (for 2 partners), and trial results might very well have indicated that the tenofovir gel is not effective in preventing HIV.

Besides these plausible sexual behavior patterns, the ratio between adherence on risky and non-risky encounters impacts the relative risk. For example, if we were to compare women with serial monogamy, then 44% adherence for unprotected and 78% adherence for protected contacts (i.e., 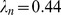 and 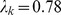) implies a relative risk of 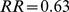. However, the same overall adherence of 70% could be obtained if we were to assume that 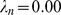 and 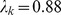 (i.e., 0 out of the 18 high-risk contacts and 63 out of the 72 low-risk contacts covered by gel use) or 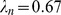 and 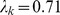 (i.e., 12 out of the 18 high-risk contacts and 51 out of the 72 low-risk contacts covered), yielding relative risks of 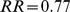 and 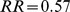, respectively. In other words, an overall adherence of 70% can imply very different relative risks depending on whether gel use is more likely for high- or low-risk contacts.

**Figure 2:**
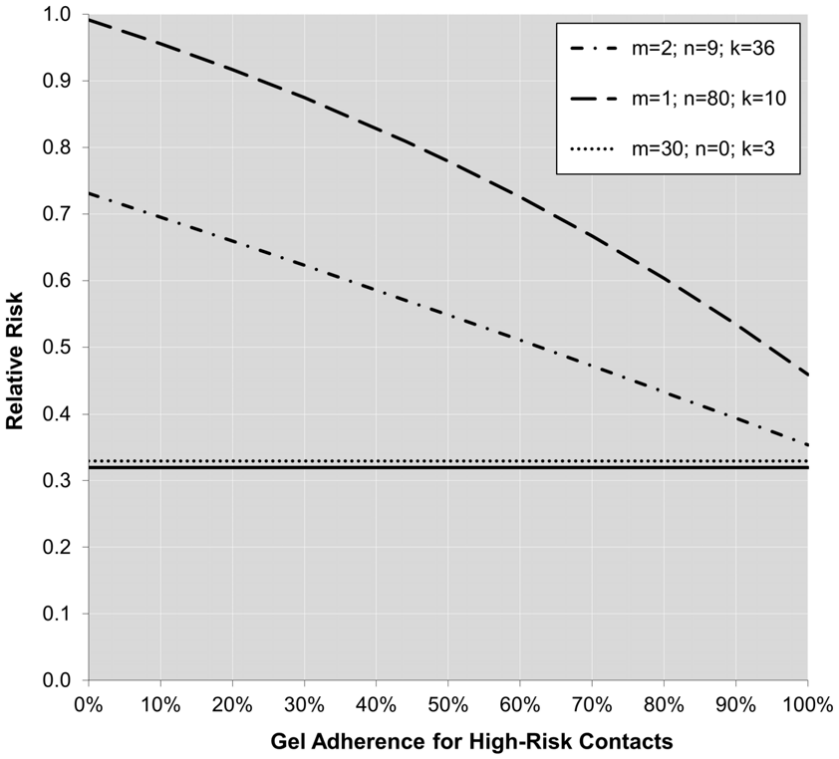
Relative infection risk as a function of adherence for high-risk contacts under the cumulative probability model for three different behavior patterns (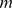 **=  number of partners, 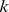 =  number of contacts per partner with condom use, 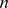 =  number of contacts per partner without condom use), assuming a true per-contact relative risk of 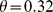 and 100% adherence for low-risk contacts.**

**Figure 3:**
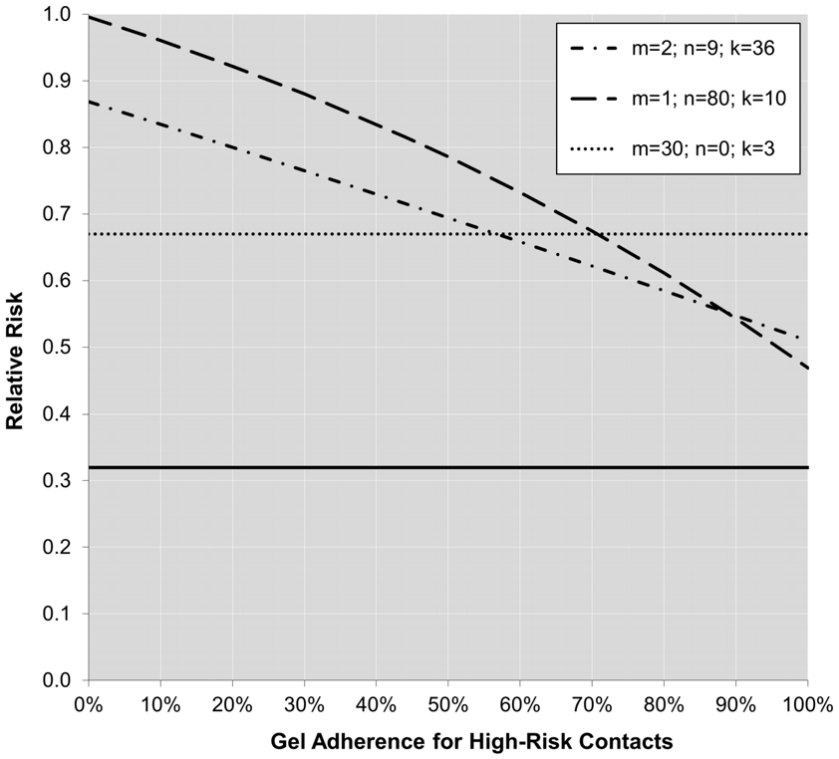
Relative infection risk as a function of adherence for high-risk contacts under the cumulative probability model for three different behavior patterns (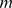 =  number of partners, 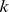 =  number of contacts per partner with condom use, 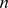 =  number of contacts per partner without condom use), assuming a true per-contact relative risk of 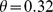 and 50% adherence for low-risk contacts.

Finally, not only is there a direct relationship between adherence and sexual behavior patterns with the relative risk of infection, also the relationship between adherence and the relative risk depends on the behavior pattern of the women. Figures 2 and 3 show how the cumulative relative risk changes as a function of adherence for high-risk contacts (i.e., 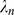) for the three different behavior patterns described earlier if we assume either 100% (i.e., 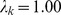) or 50% (i.e., 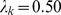) adherence for low-risk contacts, respectively. The solid horizontal line drawn at 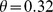 reflects the per-contact effectiveness (i.e., the true method effectiveness) of the gel in reducing the infection risk.

Figures 2 and 3 illustrate several points. First of all, an increasing level of adherence in the treatment group leads to a cumulative relative risk that approaches θ. However, the influence of adherence clearly depends on the behavior pattern (i.e., the lines are not parallel). Moreover, the relationship between adherence and the cumulative relative risk is not necessarily linear. Finally, it is important to note that even under perfect adherence the cumulative relative risk does not reach 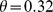, since the cumulative relative risk also depends on the sexual risk behavior patterns (a principle illustrated in Figure 1, where the cumulative relative risk approaches 1 with an increase in coital frequency regardless of adherence). Therefore, the cumulative relative risk will essentially always underestimate the true per-contact relative risk (i.e., true method effectiveness of the gel).

The results from this model and the illustrations based on the CAPRISA data therefore suggest that (a) neither the overall observed incidence rate ratio nor the observed ratio among highly adherent women in a trial reflect the true method effectiveness, (b) the secondary adherence analyses in PrEP trials oversimplify the complex interplay between adherence levels and behavior patterns, and (c) conclusions about whether or not an HIV prevention methods works can be markedly different in samples with different behavioral patterns (e.g., higher coital frequency, more partners, different levels of adherence), *regardless of the methods true effectiveness*. This could explain recent apparently contradictory trial results in this field [2], [3], [4], [5].

## Discussion

Curbing the spread of HIV remains one of the most pressing health issues to date. Microbicides and other biomedical HIV prevention measures may play an important role in this endeavor. The CAPRISA trial (and other recent trials examining early treatment and pre-exposure chemoprophylaxis interventions) [2], [34] may mark a turning point in HIV prevention research. However, we believe that the analyses conducted in PrEP trials so far have not accounted for the complex interplay between adherence, sexual risk behavior patterns, and HIV infection rates. By developing a cumulative probability model of HIV infection (e.g., [23]–[26]) to analyze HIV prevention trial results, and using data from the CAPRISA trial and several meta-analyses to illustrate the principles and implications, this paper has shown that variable risk behavior and adherence patterns in the study sample are likely to impact the primary trial conclusions about the efficacy/effectiveness of an HIV prevention measure, as well as the results of secondary adherence analyses intended to identify the impact of the gel under poor, intermediate, and optimal use. Although some of the parameters in our model had to be estimated, this leaves the principles illustrated unaffected. These findings have a range of possible implications.

First of all, the cumulative probability model revealed how adherence on high- versus low-risk contacts, condom use, the frequency of sexual contacts and the number of partners directly influence the cumulative relative risk of infection. The effects of variable behavioral patterns on the relative risk of infection can be substantial, as we illustrated, for example, by comparing the findings in the CAPRISA trial (i.e., 39% risk reduction) with the expected risk reduction if women had been somewhat more sexually active and condom usage rates were lower (i.e., a risk reduction of 21% to 26%). This finding implies that in HIV prevention trials where the sexual risk behavior variables are generally higher (e.g., due to summarizing behavior over longer follow-up periods or because of the selection of high-risk individuals), where condom use rates or adherence overall is lower, or where adherence is proportionally lower on high- versus low-risk encounters, the observed relative risk reductions will be smaller (i.e., more biased towards the null-hypothesis) *regardless* of the true effectiveness of the prevention method tested. Not only does this affects how highly trial results are being valued (e.g., trial implications are rather different when the reported risk reduction is 54%, 39%, or 21%), but this could also have a notable impact on the power to detect a significant treatment effect (computations done but not shown here). Not accounting for these issues could explain why the CAPRISA results could not be replicated in the VOICE study (among higher-risk women), or why oral PrEP has been effective in some studies but not in others [2], [3], [4], [5]. We therefore recommend researchers of previous trials to redo their analyses and control for these (time-varying) effect modifiers where possible, and future trials to start taking these factors into account when conducting sample size computations. Since the sexual behavior of the women tends to be strongly impacted by their participation in the trial, and adherence (ratios) may be unknown prior to the trial, confirming sample size calculations during early interim analyses is recommended.

Moreover, since the observed risk reduction is a combination of sexual behavior, adherence, and the true method effectiveness of the HIV prevention method tested, the results of a trial in one setting cannot be directly generalized to other settings where the target population may have very different risk behavior patterns. In fact, because of the adherence support, behavioral measurements, participant reimbursements, and the provision of condom counseling (which appears to be highly effective in all trials we examined), the behaviors of women participating in microbicide trials are very different from the behaviors they displayed prior to the trial (e.g., condom use percentages double, coital frequency and number of partners decline). Consequently, the risk reductions observed in trials are not suitable for estimating the actual impact of the gel on the HIV pandemic if disseminated on a large scale. A more accurate estimate of the potential impact of the large-scale implementation of an effective HIV prevention method could be obtained by fitting a mathematical model similar to the one we presented in this paper to identify the true method effectiveness (i.e. the reduction in the per-contact infection risk). This true method effectiveness can then be combined with ‘real-life’ data on sexual behavior to model the expected impact of large-scale implementation for several average adherence and retention levels for low- and high-risk sexual encounters. These results in turn offer targets for trials trying to identify the most cost-effective adherence support programs, targets that may vary considerably depending on the sexual risk behavior patterns in the at-risk group.

In addition to the primary analyses in microbicide trials, secondary adherence analyses are important for estimating the impact of an HIV prevention measure under variable levels of use, and for estimating the true method effectiveness of a prevention method. The current findings suggests that the results from such adherence analyses need to be reexamined because the relationship between adherence and the observed risk reduction is likely to be obscured by the sexual behavior patterns in the sample. We cannot, for example, be certain whether the risk reductions reported by the CAPIRSA authors for the different adherence levels that were averaged over high- and low-risk encounters (i.e., 54%, 38% and 28% for adherence levels of >80%, 50%-80%, and <50%, respectively) reflect the changing effectiveness of the gel as a function of adherence, or whether this relationship is (at least partially) the result of different sexual behavior patterns between women with different adherence levels, or because of co-varying ratios of adherence on high- versus low-risk encounters (e.g., with lower average adherence, gel use becomes less likely on high-risk compared with low-risk encounters). In fact, Karim and colleagues report that “women with the highest gel adherence tended to have the lowest coital frequency” (pp. 1172) [3], which could partially explain the larger treatment effects observed among high adherers.

Finally, the current approach used to identify the true method effectiveness of an HIV prevention method seems inadequate. Presently, the observed risk reduction under optimal adherence (e.g., >80%) seems to be used for that purpose. However, as shown in this paper, the cumulative relative risk among highly adherent participants still only reflects the method effectiveness of an HIV prevention method as applied in a particular sample after displaying a certain sexual behavior pattern for a particular time period. Even under 100% adherence, the observed relative risk will underestimate the per-contact relative infection risk (see Figure 2). Therefore, the cumulative relative risk is not an inherent property of the effectiveness of the intervention method itself, but reflects how well the method works in a particular context. On the other hand, the per-contact relative risk is a direct reflection of the true method effectiveness and does not depend on the particular sample of women included in the trial.

### Reservations and limitations

The mathematical model used in the present paper represents a simplified abstraction of a more complicated reality. We did not, for example, include whether sexual partners where circumcised or treated for HIV, or other factors proposed to influence the per-contact infection risk [e.g., 26, 35], since these data were unavailable. Moreover, we used the average adherence and sexual behavior patterns of women in CAPRISA. Ideally, person-level, time-stamped data would have been available for all of the variables. However, such data was not available to us nor does it seem feasible for study authors to collect such detailed information. A recent comparison between aggregate- versus individual-level data on sexual behavior indicated, however, that rather accurate estimates of HIV risk can be obtained even with relatively simple aggregate data collection techniques [36].

Applying the model to the CAPRISA trial and estimating the true method effectiveness was necessarily based on some data assumptions. For example, the ratio of adherence for low- versus high-risk encounters had to be based on the findings from three other trials ([30]–[32]) that reported this information. If, instead of assuming a ratio of 1.77 (i.e., 78% adherence for low- and 44% for high-risk contacts), we assume that adherence was approximately the same for low- and high-risk contacts (i.e., 12 out of the 18 high-risk and 51 out of the 72 low-risk contacts covered by the gel), then we obtain an estimate of 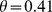, a 59% relative risk reduction per contact. If, on the other hand, we assume a similarly large deviation in the other direction (i.e., a ratio of 2.4, with 6 out of the 18 high-risk and 51 out of the 72 low-risk contacts covered by the gel), we obtain an estimate of 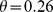, a 74% risk reduction per contact. Besides that these points illustrate that adherence ratios can have a notable impact on conclusions about method effectiveness, they also illustrate the importance of assessing behavioral data in HIV prevention trials in great detail. Regarding the current estimate of 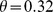, it is unlikely that the adherence ratio in CAPRISA deviated this much from these other studies. In general, several other sensitivity analyses indicated that the estimated method effectiveness of the tenofovir microbicide appears to be a relatively robust finding.

One additional aspect of our analyses with the CAPRISA data warrants some further discussion. In order to match up the remarkably high seroconversion rates in the CAPRISA study (approximately 9.1 cases per 100 women-years in the control group) with the (low-risk) sexual behavior patterns (i.e., few partners, high condom use, low coital frequency), a very high per-contact infection risk (i.e., 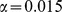) had to be assumed for non-condom contacts. This value substantially exceeds the male-to-female per contact infection risk for low-income countries in the absence of commercial sex exposure that was recently found in a meta-analysis (mean [95% CI] 0.003 [0.0014-0.0064]) [18], and so does the HIV incidence rate when compared with national surveys in South Africa (1.0–2.2 cases per 100 women-years for 15–49 year olds) [37]. Three model variables could potentially explain this finding: (1) sexual behaviors were severely underreported and/or condom use was severely overreported, (2) the prevalence of HIV in the population of male partners was much higher, or (3) the placebo gel increases the risk of infection. Although the first two of these explanations are plausible, the figures would need to deviate to such an extent (e.g., 5 times as many partners or 5 times as many sexual encounters per partner) that an additional explanation is needed. Possibly, the sexual cleansing practices that Karim and colleagues observed among the women in the region substantially increased the per-contact infection risk [38]. However, regardless of the specific value we assume for per-contact infection risk, the general principles illustrated remain unchanged.

## Conclusions

The current study suggests that sexual behavior and adherence patterns among participants in HIV prevention studies impact the relative risk of infection and secondary adherence analyses. Taking these behaviors into account may improve study design, guide data collection, help to identify effective prevention methods and the impact of variable adherence levels, and contribute to resolving current debates about contradictory HIV prevention trial results [39].
